# Incidence and mortality of necrotizing fasciitis in The Netherlands: the impact of group A Streptococcus

**DOI:** 10.1186/s12879-021-06928-5

**Published:** 2021-12-06

**Authors:** Femke Nawijn, Brechje de Gier, Diederik A. H. Brandwagt, Rolf H. H. Groenwold, Jort Keizer, Falco Hietbrink

**Affiliations:** 1 Department of Surgery, University Medical Center Utrecht, Utrecht, The Netherlands; 2 Center for Infectious Disease Control, National Institute for Public Health and the Environment (RIVM), Utrecht, The Netherlands; 3 Department of Infectious Diseases, Public Health Service (GGD) Region Utrecht, Utrecht, The Netherlands; 4 Department of Clinical Epidemiology, Leiden University Medical Center, Leiden, The Netherlands; 5 Department of Surgery, Sint Antonius Hospital, Utrecht, The Netherlands

**Keywords:** Necrotizing fasciitis, Necrotizing soft tissue infection, Incidence, The Netherlands, Mortality, Health care burden

## Abstract

**Background:**

Little is known about the exact incidence of necrotizing soft tissue infections. The few incidences reported in international literature are not directly relatable to the Netherlands, or other European countries, due to geographic heterogeneity in causative micro-organisms involved. This resulted in the aim of this study to map the incidence, mortality rate and hospital course of necrotizing fasciitis infections in the Netherlands to gain insight in the incidence of necrotizing fasciitis in the Netherlands and the associated mortality and health care burden.

**Methods:**

This nationwide retrospective database study used three distinct data sources to map the incidence of necrotizing fasciitis in the Netherlands between 2014 and 2019, being data from the Dutch Hospital Data (DHD) foundation, data from Osiris-AIZ, which is a database of notifiable diseases managed by regional Public Health Services (GGD) and the National Institute for Public Health and the Environment (RIVM), and previously published studies on necrotizing fasciitis conducted in the Netherlands.

**Results:**

The incidence of necrotizing fasciitis in the Netherlands is estimated to be approximately 1.1 to 1.4 cases per 100,000 person years, which corresponds to 193–238 patients per year. Of all necrotizing fasciitis infections, 34 to 42% are caused by the group A Streptococcus. Annually, 56 patients die as a result of a necrotizing fasciitis infection (mortality of 23–29%) and 26 patients undergo an amputation for source control (11–14%). Patients stay a mean of 6 to 7 days at the intensive care unit and have a mean hospital length of stay of 24 to 30 days.

**Conclusion:**

The combination of nationwide databases provides reliable insight in the epidemiology of low-incidence and heterogenic diseases. In the Netherlands, necrotizing fasciitis is a rare disease with group A Streptococcus being the most common causative micro-organism of necrotizing fasciitis. The prior Dutch cohort studies on necrotizing fasciitis report slightly higher sample mortality rates, compared to the population mortality. However, necrotizing fasciitis remain associated with substantial morbidity and mortality, risk at amputation and health care burden characterized by prolonged ICU and hospital stay.

## Background

Necrotizing fasciitis is a rare, bacterial infection of the fascia, characterized by rapidly progressive soft tissue necrosis and (impending) sepsis [1]. Umbrella terms such as necrotizing soft tissue infections (NSTI) or severe necrotizing soft tissue disease (SNSTD) are becoming more commonly used terms to denote necrotizing fasciitis, but also refer to other necrotizing infectious diseases such as gas gangrene, necrotizing cellulitis, necrotizing myositis and combination diseases. However, necrotizing fasciitis remains the most notorious and most common form of a NSTI [2, 3]. The mortality rate of these NSTIs declined by almost half since the beginning of the twenty-first century compared to the twentieth century, however, the mortality rate remained stable around 20% during the past two decades [4]. Achieving further decrease in mortality rates still seems to be limited by delay in diagnosis and therefore treatment [2, 4, 5]. Timely diagnosis is especially difficult due to the low incidence of necrotizing fasciitis and its heterogeneous presentation [6–8].

These same factors of heterogeneity and low incidence also hinder the conduct of sufficiently powered and generalizable studies to gain knowledge into how timely diagnosis and treatment can be improved.

On one hand, available studies on necrotizing fasciitis are often small single or multicenter (retrospective) cohort studies performed by institutes with particular interest in the disease. This might introduce selection bias with potentially relatively higher incidences and lower mortality rates (due to extra awareness and special interest).

On the other hand, to gain true insight in the incidence of this rare disease, nationwide studies might provide more accurate information, however, most of these studies are based on only one nationwide database, most often an hospital imbursement database, and are mostly conducted in Asia or the United States [8–10]. It is difficult to interpreter such large, nationwide, finance-based databases as, for example, there is a risk of over- or underreporting and heterogeneity in registration of the data. There are a few studies available that have reported the incidence of necrotizing fasciitis: 0.86 cases per 100,000 person years in South Korea, 1.3 cases per 100,000 person years in New Zealand and 4 to 10.3 cases per 100,000 person years in the United States [6–8, 11]. These incidence rates are not directly applicable to the Netherlands, due to known geographic heterogeneity in causative micro-organisms involved in necrotizing fasciitis infections and the corresponding differences in, for example, age distribution and mortality [12–18]. For example, in North and South America (methicillin-resistant) S. aureus is the most common causative organism of skin and soft tissue infections (including necrotizing fasciitis), while this incidence is in Europe significantly lower [19]. Therefore, the incidence of necrotizing fasciitis in the Netherlands remains uncertain, resulting in the aim of this study to map the incidence, mortality rate and hospital course of necrotizing fasciitis infections in the Netherlands by using different types of nationwide databases, a hospital imbursement database and a notifiable diseases database, and previous published cohort studies conducted on necrotizing fasciitis in the Netherlands to enable correcting for over- and underreporting of each source.

## Methods

To map the nationwide incidence of necrotizing fasciitis in the Netherlands, three distinct data sources were used, being (1) data from the Dutch Hospital Data (DHD) foundation, (2) data from Osiris-AIZ, which is a national database of notifiable diseases managed by regional Public Health Services (GGD) and the National Institute for Public Health and the Environment (RIVM), and (3) previously published studies on necrotizing fasciitis conducted in the Netherlands (Table 1) [12–14].

**Table 1:** Data sources used to gain insight in the incidence of necrotizing soft tissue infection in the Netherlands

Database	Obtained information	Period
*Dutch Hospital Data (DHD) Foundation*		
	All necrotizing fasciitis cases registered based on International Classification of Disease (ICD) 9 and 10 diagnosis and procedural codes in this nationwide registry linked to hospital imbursements systems, including data on mortality rates, amputation rates, number of operative procedures, length of intensive care unit stay and length of hospital stay	January 2014–January 2020
*National Institute for Public Health and the Environment (RIVM)*		
Osiris-AIZ	Nationwide registry containing all cases of notifiable diseases registered anonymously by the regional public health services (GGD), including GAS necrotizing fasciitis cases and the associated mortality per age category	January 2011–January 2020
Regional Public Health Services (GGD) of the Utrecht region	Number of registered Group A Streptococcal necrotizing fasciitis cases within the region of the GGD Utrecht, including year of notification, patient’s age at time of diagnosis and notifying hospital	January 2009–July 2016
*Published Dutch retrospective databases*		
Nawijn et al. (2019)	Patients with necrotizing fasciitis presenting to two different hospitals within the region of Utrecht (one academic medical center and one large peripheral teaching hospital)	August 2002–September 2016
Van Stigt et al. (2016)	Patients with necrotizing fasciitis presenting to four different hospitals within the region on Gelderland (one academic medical center and three peripheral teaching hospitals)	January 2003–December 2013
Suijker et al. (2020)	Patients with necrotizing soft tissue infection presenting to a large academic medical center within Amsterdam	2000–2012

The DHD foundation registers data from all (both peripheral and academic) hospitals with an emergency department in the Netherlands by using a standardized diagnosis- and procedure thesaurus, directly linked to hospital imbursement systems, with as aim to support health care quality, decision-making and management. To ensure quality and accuracy of their imputed data, quality checks and standards are in place. From the DHD foundation, the number of registered necrotizing fasciitis patients (including Fournier gangrene, and based on the DHD diagnosis thesaurus combined with international classification of Diseases (ICD) 9 and 10 codes (ICD 9: 728.86 and 608.83, ICD 10: M72.6 and N49.3) and procedure codes, protocol available upon request at DHD foundation) within the Netherlands between January 1st, 2014 and December 31st, 2019 were obtained, including the frequency in which mortality and amputations occurred, the number of operative procedures, and the length of hospital and intensive care (ICU) stay. Importantly, the data from the DHD foundation might overestimate the incidence, considering that transferred patients might be registered in duplicate in the database (this cannot be corrected for, since the data supplied by the DHD foundation was aggregated and pseudo-anonymized).

**Fig. 1:**
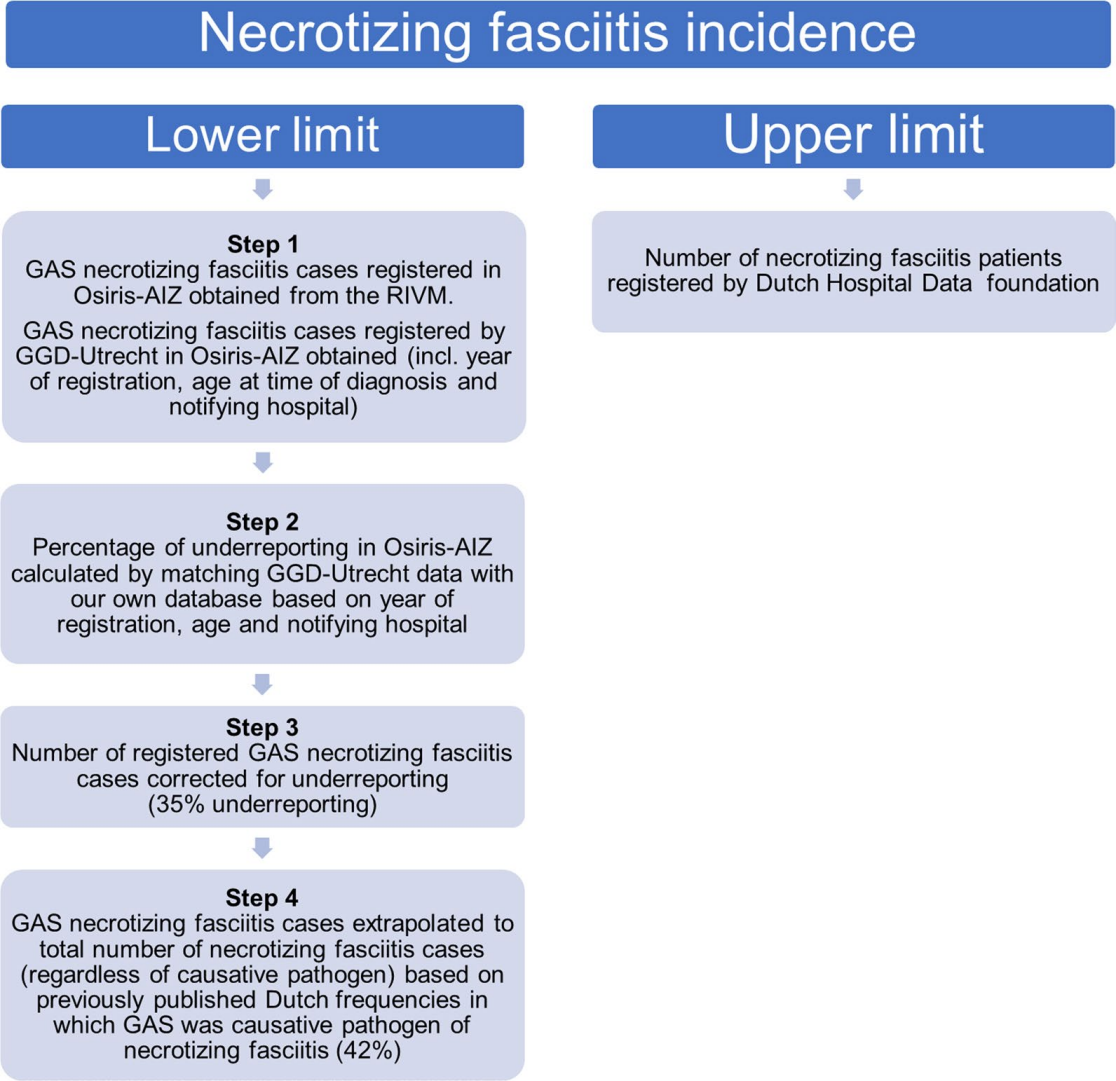
Methods used to map incidence of necrotizing fasciitis in the Netherlands. *GAS*  Group Streptococcal, *GGD*  Regional Public Health Services, *Osiris-AIZ*  nationwide database of notifiable infectious diseases managed by GGD and RIVM, *RIVM*  National Institute for Public Health and Environment

In the Netherlands, invasive group A streptococcal (GAS) infections are notifiable diseases since 2008 and therefore have to be reported to and registered by the GGD in the national Osiris-AIZ database managed by the RIVM. All other micro-organisms causing necrotizing fasciitis do not have to be reported to the RIVM. For this study, all reported GAS necrotizing fasciitis cases between January 1st, 2011 and December 31st, 2019 were requested, including age distribution of the patients and registered mortality. The registered mortality in Osiris-AIZ is commonly underreported since it is not obligatory for health care workers to report if a patient died after the notification of the infection has already been made. Furthermore, the GGD of the region Utrecht was asked for the registered cases of GAS necrotizing fasciitis within the same time period, including year of notification, patient’s age at time of diagnosis and notifying hospital. These extra variables are not registered within the national Osiris-AIZ database. By obtaining these variables, the patients reported to the GGD in the region Utrecht could be matched to a previous by our own study group published database of necrotizing fasciitis patients between January 2002 and August 2016 performed at two of the hospitals within the same region (an academic hospital and a large peripheral hospital) to estimate the (in)completeness of the Osiris-AIZ database (step 2 of Fig. 1) [12]. This study was conducted in accordance with relevant guidelines and regulation and was approved by the institutional review board of both centers (University Medical Center Utrecht and St. Antonius Hospital), which provided a waiver of informed consent. This incompleteness was anticipated, since even though GAS necrotizing fasciitis is a notifiable disease, it is likely that in some cases the GGD was not notified. During this matching process, six patients were identified that were reported to the GGD of the region Utrecht, but were not included in our own database, and fourteen patients with GAS necrotizing fasciitis were included in our own database but were not registered by the GGD. This resulted in a total of 40 registered, unique cases between 2011 and 2016 at the two study hospitals. To correct for this underreporting, the reported GAS necrotizing fasciitis cases were extrapolated using the estimated percentage of incompleteness of the Osiris-AIZ database (14/40 not reported to the GDD; 35% underreporting, 95% confidence interval (CI) 20–50%).

Due to the potential under- and overreporting within the different databases, the choice was made to present the Dutch incidence as an extreme estimate of the actual incidence of necrotizing fasciitis in the Netherlands. The upper limit of the estimated incidence is based on the data from the DHD foundation. The lower limit is based on the data from Osiris-AIZ corrected for the estimated incompleteness of the database and extrapolated to all necrotizing fasciitis cases (regardless of the causative micro-organism) based on all previous published studies reporting frequencies in which GAS was the causative micro-organism of necrotizing fasciitis in the Netherlands (overall 42%, 95% CI 34–49%) (Step 3 and 4, Fig. 1) [12–14].

To illustrate the age distribution of reported GAS necrotizing fasciitis cases, the age distribution was standardized to the age distribution in the Netherlands using the population age distribution from the Dutch Central Bureau for Statistics, illustrating the incidence of GAS necrotizing fasciitis cases per 100,000 person years per age category [20]. Dichotomous variables were analyzed using the Fisher’s exact test. For all analyses, a two-sided *p*-value < 0.05 was considered statistically significant. Data were analyzed using STATA (StataCorp. 2013. Stata Statistical Software: Release 13. College Station, TX: StataCorp LP).

## Results

### Estimated incidence

**Fig. 2:**
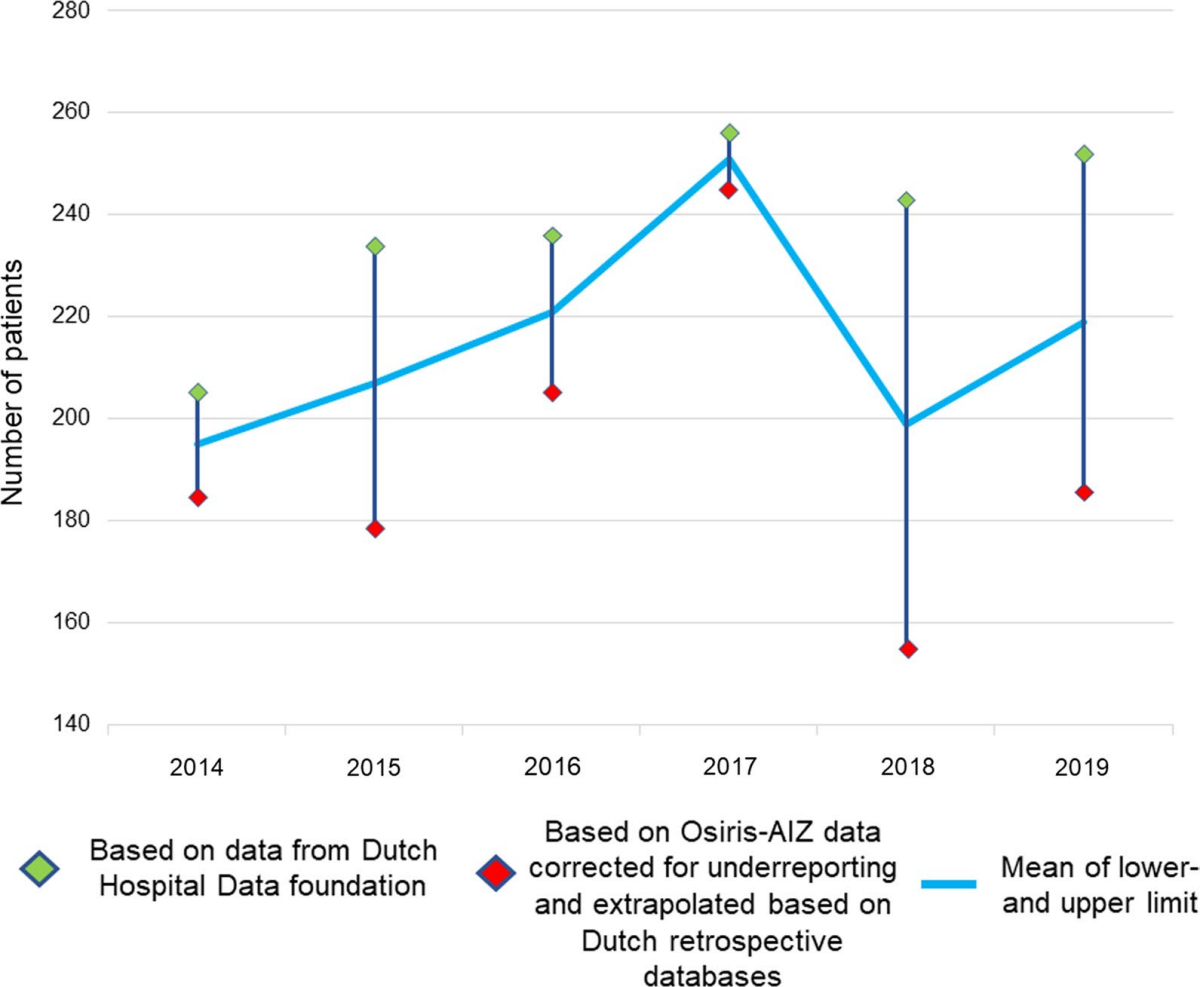
Incidence of necrotizing fasciitis in the Netherlands (2014–2019)

**Fig. 3:**
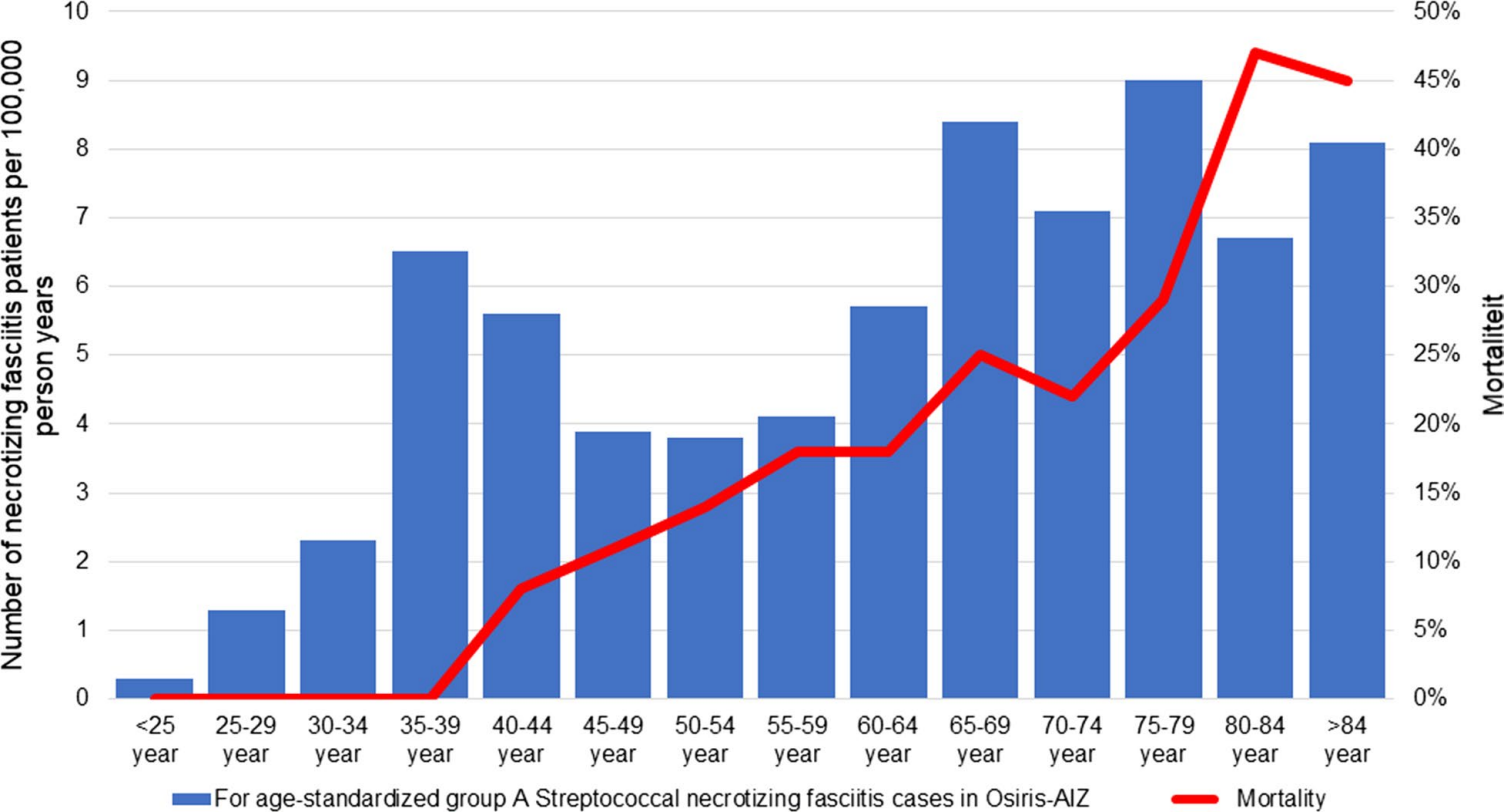
Incidence of group A Streptococcal fasciitis necroticans patients reported in Osiris-AIZ per age category and associated mortality (2009–2019)

The incidence of necrotizing fasciitis in the Netherlands is estimated to be approximately 1.1 to 1.4 cases per 100,000 person years, which corresponds to 193–238 patients per year in the Netherlands (Table 2 and Fig. 2). Per year, 81 of these patients had necrotizing fasciitis caused by GAS (34–42%), with a peak in incidence within the age category of 65 years and older (Table 3 and Fig. 3). The DHD foundation data showed that most necrotizing fasciitis patients were treated in peripheral hospitals (81% of all registered patients), however, it is with the currently available information unknown how many patients of those patients were transferred to academic (in case of critical illness) or burn centers (in case of extensive reconstructions).

**Table 2:** Incidence, mortality and health care burden of necrotizing fasciitis in the Netherlands (2014–2019)

Year	Number of necrotizing fasciitis cases^a^	Mortality^b^	Amputations^b^	Number of operative procedures^b^ (average per patient)	Length of ICU stay in days^b^ (average per patient)	Length of hospital stay in days^b^ (average per patient)
2014	185–205	46 (22–25%)	19 (9–10%)	346 (1.7–1.9)	1029 (5–6)	4600 (22–25)
2015	179–234	61 (26–34%)	30 (13–18%)	416 (1.8–2.3)	1122 (5–6)	5967 (26–33)
2016	205–236	51 (22–25%)	23 (10–11%)	429 (1.8–2.1)	1463 (6–7)	5945 (25–29)
2017	245–256	62 (24–25%)	24 (9–10%)	522 (2.0–2.1)	1394 (5–6)	6097 (24–25)
2018	155–243	62 (26–40%)	42 (17–27%)	470 (1.9–3.0)	1416 (6–9)	5589 (23–36)
2019	186–252	53 (21–28%)	19 (8–10%)	468 (1.9–2.5)	1420 (6–8)	5885 (23–32)
Total	1155–1426	335 (23–29%)	157 (11–14%)	2651	7844	34,083
Yearly average	193–238	56 (23–29%)	26 (11–14%)	442	1307	5681
Average per patient	NA	NA	NA	1.9–2.3	6–7	24–30

*ICU* intensive care unit

Data sources: ^a^Lower limit based on cases registered in Osiris-AIZ, on cases registered by the GGD Utrecht combined with previous Dutch retrospective databases, upper limit based on data from the Dutch Hospital Data (DHD) Foundation

^b^DHD Foundation

**Table 3:** Incidence and mortality of (non-) Group A Streptococcal necrotizing fasciitis in the Netherlands (2014–2019)

Year	Number of necrotizing fasciitis cases^a^	GAS necrotizing fasciitis cases registered in Osiris-AIZ^b^	Number of GAS necrotizing fasciitis cases corrected for underreporting^c^	Number of non-GAS necrotizing fasciitis cases^d^	Mortality^e^	Mortality of GAS necrotizing fasciitis registered in Osiris-AIZ^b^	Mortality of GAS necrotizing fasciitis corrected for underreporting^c^	Mortality of non-GAS necrotizing fasciitis^d^
2014	185–205	51	78 (38–42%)	107–127 (58–62%)	46 (22–25%)	11	17 (22%)	29 (23–27%)
2015	179–234	49	75 (32–42%)	104–159 (58–68%)	61 (26–34%)	5	8 (11%)	53 (33–51%)
2016	205–236	56	86 (36–42%)	119–150 (58–64%)	51 (22–25%)	14	22 (26%)	29 (19–24%)
2017	245–256	67	103 (40–42%)	142–153 (58–60%)	62 (24–25%)	8	12 (12%)	50 (33–35%)
2018	155–243	42	65 (27–42%)	90–178 (58–73%)	62 (26–40%)	7	11 (17%)	51 (29–57%)
2019	186–252	51	78 (31–42%)	108–174 (53–69%)	53 (21–28%)	9	14 (18%)	39 (22–36%)
Total	1155–1426	316	485 (34–42%)	670–941 (58–66%)	335 (23–29%)	54	84 (17%)	251 (27–37%)
Yearly average	193–238	53	81 (34–42%)	112–157 (58–66%)	56 (23–29%)	9	14 (17%)	42 (27–37%)

GAS = Group A Streptococcus

Data sources: ^a^Lower limit based on cases registered in Osiris-AIZ, on cases registered by the GGD Utrecht combined with previous Dutch retrospective databases, upper limit based on data from the Dutch Hospital Data (DHD) Foundation; ^b^Osiris-AIZ; ^c^Number of registered cases in Osiris-AIZ corrected for underreporting by using data from the GGD-Utrecht and our own retrospective database from the same geographic region; ^d^Based on data source 1 and 3; ^e^DHD Foundation

### Mortality

**Fig. 4:**
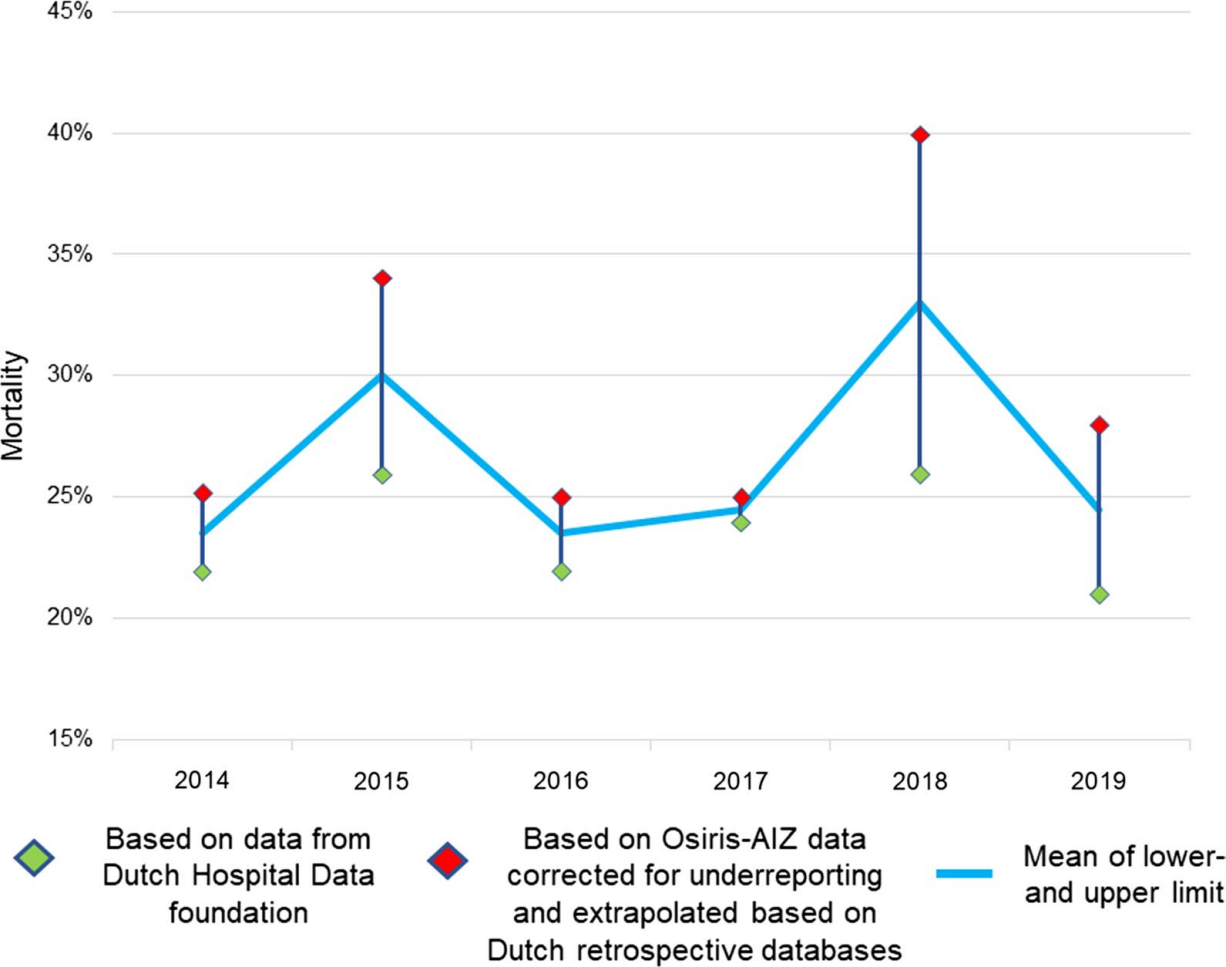
Mortality of necrotizing fasciitis in the Netherlands (2014–2019)

Between January 2014 and December 2019, a total of 335 patients died as result of necrotizing fasciitis (on average 56 patients per year), representing a mortality rate of 23–29%. During those 6 years, the incidence and mortality of necrotizing fasciitis in the Netherlands remained stable (Fig. 4). Of the 81 GAS necrotizing fasciitis patients per year, an average of 14 patients per year died as result of the infection (average mortality rate of 17%). Older age was associated with an increased risk at mortality in GAS necrotizing fasciitis patients, with a mortality rate of 46% in the age category of 80 years and older (Fig. 3). Necrotizing fasciitis patients caused by other micro-organisms than GAS (for example, polymicrobial infection with anaerobic and aerobic bacteria, *Staphylococcus aureus*, *Clostridium* spp.) had a mortality rate of 27–37% (Table 3). Based on the DHD foundation data, no difference in mortality was found between academic and peripheral hospitals (57/265 (22%) vs. 278/1161 (24%), *p* = 0.423).

### Hospital course

Dutch patients with necrotizing fasciitis undergo on average 1.9 to 2.3 operative procedures, which also includes patients who did not undergo any surgical procedures, for example due to withdrawal of care. Annually, 26 patients undergo an amputation as treatment for a necrotizing fasciitis (11–14%). Amputations were more often performed at academic hospitals compared to peripheral hospitals (48/265 in academic hospitals (18%) vs. 109/1161 in peripheral hospitals (9%), *p* < 0.001). Patients had a mean ICU stay of 6 to 7 days (incl. patients without ICU admission) and a mean hospital length of stay of 24 to 30 days*.*

## Discussion

This study is the first to investigate the incidence of necrotizing fasciitis in the Netherlands, which resulted in an estimated incidence of approximately 1.1–1.4 cases per 100,000 person years. The Dutch mortality rate of necrotizing fasciitis (23–29%) is slightly higher than the reported mortality rate of 18–21% in recent international literature from selected centers and is slightly lower than the pooled mortality rate from the three previous Dutch cohort studies (28%). The Dutch mortality rate for GAS necrotizing fasciitis (17%) is comparable to that found in other European studies on GAS necrotizing fasciitis (10–22%) [4, 12, 21, 22]. In the current study a higher mortality rate was observed in non-GAS necrotizing fasciitis patients compared to GAS necrotizing fasciitis patients. Based on previous literature, this difference in mortality might be due to the fact that non-GAS necrotizing fasciitis patients tend to be older and to have more severe and/or multiple comorbidities compared to GAS necrotizing fasciitis patients [12, 23]. Factors such as age, comorbidities (e.g. diabetes mellitus, renal failure, history of malignancy) and laboratory results upon presentation (e.g. creatinine, lactate) are frequently reported to be potential predictors for mortality in these patients [15, 24, 25]. Nonetheless, the most important, potentially modifiable predictor for mortality remains time to treatment [4]. Early recognition, followed by prompt (preferable within 6 h) and adequate surgical and antibiotic treatment is of utmost importance due to the progressive nature of the infection [2, 4]. For example, only 52% of the patients who underwent surgical treatment and adequate antibiotic treatment for their necrotizing fasciitis will have resolution of fever and some indication of lesion improvement or stability within 72 h [26]. Initiating prompt treatment is frequently hindered by a delayed diagnosis caused by a misdiagnosis upon presentation, with reported rates as high as 70%, due to its low incidence and the absence of pathognomic symptoms upon presentation [5]. To obtain prompt and acquired diagnosis and treatment, it requires a multidisciplinary approach with involvement of surgeons, medical microbiologists, pathologists, intensive care physicians, and often also plastic surgeons, infectious disease physicians, otolaryngologists, urological surgeons, and within the phase of rehabilitation also involve physiatrists [3, 27].

In the Netherlands, notable more patients underwent an amputation in an academic hospital than in a peripheral hospital, while there was no difference in mortality between both types of hospitals. The Dutch healthcare system is constructed in such a way, that it is tempting to speculate that necrotizing fasciitis patients who present to academic hospitals (primarily or secondarily) had more comorbidities, a more severely extended infection (potential due to delay in presentation) and/or had a higher degree of physiological derangement upon presentation warranting a more aggressive surgical approach to obtain source control. A previous meta-analysis showed that treatment delay does not necessarily result in a higher rate of amputations and another study was not able to find a correlate between the amputation rate and specific causative micro-organisms (GAS necrotizing fasciitis vs. necrotizing fasciitis caused by other micro-organisms), however other studies have shown that factors such as sepsis and transfer to another hospital are predictors for amputation as treatment [4, 12, 15, 28, 29].

Nowadays, there is a growing interest in the skin sparing approach for necrotizing fasciitis based on the hypothesis that it would results in less reconstructive surgeries and less wound healing complications [30]. Those advantages would contribute to a shorter hospital stay, which has been associated with a better quality of life after necrotizing fasciitis, but could also lower health care costs [30, 31]. Unfortunately, studies on the outcomes of the skin sparing technique remain scarce. One of the few studies showed that wounds can be closed earlier on and that fewer patients required skin grafts [32]. However, in most studies on the skin sparing approach the technique was mainly used during the secondary debridement in transferred patients (85%) and in patients in who the initial debridement was not performed skin sparing (32–71%), limiting conclusion to be drawn about outcomes of the approach if it is used during the initial debridement [32, 33]. Preventing mortality by performing adequate source control remains the primary goal with extra consideration for long-term function and aesthetics as secondary goals.

One of the previous mentioned advantages of the skin-sparing technique was the possibility to reduce health care costs. Currently, the exact health care costs linked to a necrotizing fasciitis infection in the Netherlands remain unknown, while costs of approximately $50,000 per patient have been reported by two prior studies (Australia and United States) [11, 34]. Mapping the Dutch health care costs is especially difficult due to the great variety of health care codes used to declare costs for these patients. For example, in the Netherlands, the DHD foundation found 483 different procedure codes declared for necrotizing fasciitis patients. This is a consequence of the fact that currently there are no codes specially for procedures performed to treat necrotizing fasciitis, which results in physicians to use many different (and frequently vague) terms to described and register the procedures. Furthermore, not all these diagnosis- and procedure codes cover the full costs entailing the treatment for necrotizing fasciitis. Unambiguously registration would undoubtedly improve research into, knowledge about and insight in health care costs related to necrotizing fasciitis.

The results should be interpreted considering the study’s limitations. First, nationwide database provide mostly general information (e.g. ICU days, hospitals days), without many disease-related details such which limb was amputated, if a patient required invasive respiratory support or which antibiotics were given. Second, the under- and overreporting in the different databases caused a certain level of uncertainty in our results. For example, the percentage of underreporting of GAS necrotizing fasciitis patients in Osiris-AIZ. Potentially, patients were not reported to the GGD or coded as streptococcal toxic shock syndrome instead of necrotizing fasciitis. Nonetheless, this is the first study introducing a method for combing nationwide databases containing necrotizing fasciitis patients, with different data sources (in this case a nationwide hospital billing database and the notifiable disease database by the National Institute for Public Health), with previously publish literature from the same geographic region to map the incidence of necrotizing fasciitis within a country. This method aimed for the highest accuracy of the estimated incidence as possible based on the available data by acknowledging the possibility of under- and overreporting within the databases and correcting for this, and by recognizing the uncertainty by providing interval estimates instead of point estimates. Therefore, in contrast to cohort studies which base their incidence on extrapolation of a sample from a specific hospital or region, this study was able to provide data on necrotizing fasciitis from the entire Dutch population using two databases which have incorporated quality checks to maintain quality and accuracy,

## Conclusion

The combination of nationwide databases provides reliable insight in the epidemiology of low-incidence and heterogenic diseases. In the Netherlands, group A streptococcal is the most common causative micro-organism of necrotizing fasciitis. Necrotizing fasciitis is still associated with substantial morbidity and mortality, risk at amputation and health care burden characterized by prolonged ICU and hospital stay. The main focus should be to further reduce mortality by improving and facilitating prompt recognition of necrotizing fasciitis, followed by reducing the morbidity and improving long-term function and quality of life.

## Data Availability

The datasets used and/or analyzed during the current study are available from the corresponding author on reasonable request.

## Abbreviations

CI: Confidence interval
DHD: Dutch Hospital Data
GAS: Group A Streptococcus
ICD: International Classification of Disease
ICU: Intensive care unit
GGD: Regional Public Health Services
NSTI: Necrotizing soft tissue infection
RIVM: The National Institute for Public Health and the Environment
SNSTD: Severe necrotizing soft tissue infection
